# Super‐High Sodium‐Ion Conductivity of Na_2.9_Sb_0.9_W_0.1_S_4_ at Low Pressures by Systematic Pressure and Temperature Treatments

**DOI:** 10.1002/smll.202600075

**Published:** 2026-05-10

**Authors:** Miriam R. Bauer, Marvin Szabo, Stefanie Dehnen

**Affiliations:** ^1^ Institute of Nanotechnology (INT) Karlsruhe Institute of Technology (KIT) Karlsruhe Germany

**Keywords:** battery materials, low pressure, sodium solid electrolytes, super‐high ion conductivity

## Abstract

Sodium‐ion solid electrolytes with high ionic conductivity at low mechanical pressures are essential for the practical implementation of solid‐state batteries. We investigated the combined influence of pressure and temperature on the conductivity of the sulfide‐based sodium electrolyte Na_2.9_Sb_0.9_W_0.1_S_4_. A series of thermo‐mechanical protocols were applied in which the pellets were first subject to high‐pressure compaction (≤ 664 MPa) and subsequently annealed at 250°C for 1 h under a constant pressure (97 MPa). Electrochemical impedance spectroscopy (EIS) was performed to monitor the ionic conductivity. The most effective protocol (664 MPa → 250°C, 97 MPa) yielded a record superionic conductivity of 44.7 mS cm^−1^ at pressures ≥ 18 MPa. After one week under constant pressure (97 MPa), and subsequent relaxation to 1.3 MPa, the conductivity remained at 13.1 mS cm^−1^. Lower‐pressure treatment also caused high conductivity (9.1 mS cm^−1^ at 1.3 MPa), demonstrating the material's robustness. Powder X‐ray diffraction confirmed a pressure‐induced transition from tetragonal to cubic modifications of Na_2.9_Sb_0.9_W_0.1_S_4_, correlating with the conductivity enhancement. The results demonstrate that systematic pressure and temperature treatments afford super‐high sodium‐ion conductivity in Na_2.9_Sb_0.9_W_0.1_S_4_ at pressures compatible with industrial battery manufacturing, highlighting its promise as a solid electrolyte for next‐generation sodium‐ion batteries.

## Graphical abstract

Systematic pressure and temperature treatment of the sodium ion electrolyte Na_2.9_Sb_0.9_W_0.1_S_4_ enabled deep insight into the effects of these parameters on the sodium conductivities and their impedance‐spectroscopic measurements, and afforded a material exhibiting a record superionic conductivity of 44.7 mS cm^−1^ at pressures down to 18 MPa and an excellent value of 13.1 mS cm^−1^ at 1.3 MPa.

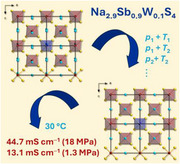

## 1 Introduction

The development of highly conductive solid‐state electrolytes (SSE) for use in all‐solid‐state batteries (ASSBs) for efficient energy storage techniques is a key challenge for the transition toward renewable energy sources. All solid‐state Na‐based batteries are regarded as next‐generation energy storage materials due to the abundance of sodium resources [1, 2]. Moreover, it was proven that particular sodium SSEs are able to compete with their lithium counterparts [3]. W‐doped Na_3_SbS_4_ is a promising SSE candidate, reaching outstanding ionic conductivities above 30 mS cm^−1^ at room temperature [4, 5]. The low‐temperature impedance study by Königsreiter et al. suggests that even higher conductivities are achievable by reducing grain‐boundary effects [6]. As the occurrence of internal pores and grain boundaries can be tuned by controlling the processing conditions during SSE pellet making [7, 8, 9], the conductivity of solid‐state electrolytes depends strongly on temperature and pressure applied during the generation of the pellet [10]. Consequently, temperature and pressure are also parameters of high significance during impedance measurements. However, partially incomplete or inconclusive reporting of measurement parameters in the literature (Table 1), makes the comparison of conductivity results difficult [11].

**TABLE 1:** Overview of reported compounds with their conductivity *σ* at 25°C and corresponding reported parameters for pressures *p* at pellet formation and impedance measurements, in comparison with this work.

Sample	*σ*_25°C_ (mS cm^−1^)	*p* at pellet formation (MPa)	*p* during impedance measurement (MPa)	Refs.
Na_2.9_Sb_0.9_W_0.1_S_4_	41 ± 8	380	—	[4]
Na_2.88_Sb_0.88_W_0.12_S_4_	32	1080	—	[5]
Na_2.9_P_0.9_W_0.1_S_4_	13 ± 3	380	—	[4]
*c*‐Na_3_PS_4_	0.46	—	—	[12]
*t*‐Na_3_SbS_4_	3	400	—	[13]
*c*‐Na_3_SbSe_4_	3.7	∼300	—	[14]
Na_11_Sn_2_PS_12_	1.4	—	—	[15]
Li_2_S‐P_2_S_5_	17	94	94	[16]
Na_2.9_Sb_0.9_W_0.1_S_4_	44.7	664	18	This work
Na_2.9_Sb_0.9_W_0.1_S_4_	13.1	487	1.3	This work

Moreover, the pressure applied in the conductivity measurement indicates the applicability of SSEs for batteries. High stack pressures on ASSBs are accompanied with challenges concerning the material structure and technical implementation [17, 18]. In order to develop pressure‐insensitive batteries, SSEs hence need to reach sufficient conductivity at low pressures. So far, no studies have reported the outstanding low‐pressure performance of Na_2.9_Sb_0.9_W_0.1_S_4_ at only 1.3 MPa, which we are going to present herein.

## 2 Results and Discussion

**FIGURE 1:**
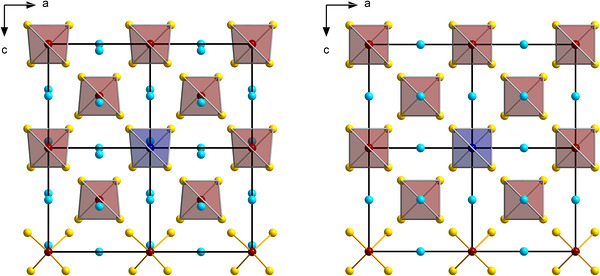
Structural diagrams (2 × 2 × 2 supercells) of tetragonal *t*‐Na_2.9_Sb_0.9_W_0.1_S_4_ (left) and cubic *c*‐Na_2.9_Sb_0.9_W_0.1_S_4_ (right) viewed along the crystallographic *b* axis. Ten [SbS_4_]^3−^/[WS_4_]^2−^ units are emphasized by polyhedral representation. One randomly selected polyhedron out of ten is indicated as being W‐centered according to the doping concentration. Color code: Sb, red, Na, turquoise, W, blue, S, yellow.

In this study, we focused on the thermomechanical property changes in the tungsten‐substituted thioantimonate, Na_2.9_Sb_0.9_W_0.1_S_4_. Like the parent compound, Na_3_SbS_4_, the solid can adopt two modifications, a cubic and a tetragonal one, denominated as *t*‐Na_2.9_Sb_0.9_W_0.1_S_4_ or *c*‐Na_2.9_Sb_0.9_W_0.1_S_4_, respectively (Figure 1).

**SCHEME 1:**
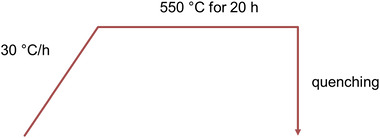
Temperature profile for synthesis of sulfide‐based solid electrolytes Na_3_SbS_4_ and Na_2.9_Sb_0.9_W_0.1_S_4_. Starting and end points are room temperature.

We first set out to establish a definition of a benchmark. For this, we synthesized known sodium solid electrolytes Na_3_SbS_4_ and Na_2.9_Sb_0.9_W_0.1_S_4_ according to the reported temperature program indicated in the Experimental Section (Scheme 1) [4], and analyzed the phases using powder X‐ray diffractometry (PXRD) as well as electrochemical impedance spectroscopy (EIS). According to PXRD, we obtained the tetragonal phases *t*­Na_3_SbS_4_ and *t*‐Na_2.9_Sb_0.9_W_0.1_S_4_ (Figure S1). Temperature‐dependent EIS was measured on both materials as described in the Supporting Information. The unsubstituted *t*­Na_3_SbS_4_ shows a room temperature (RT) conductivity at 389 MPa of 0.6 mS cm^−1^ (0.11 eV), while *t*‐Na_2.9_Sb_0.9_W_0.1_S_4_ exhibits 8.3 mS cm^−1^ (0.06 eV) at the same pressure (Figure S2). The measured values were generally lower than the reported values of 3 mS cm^−1^ [13] for *t*­Na_3_SbS_4_ and 41 ± 8 mS cm^−1^ [4] for *c*‐Na_2.9_Sb_0.9_W_0.1_S_4_. Since there is no reported value for *t*‐Na_2.9_Sb_0.9_W_0.1_S_4_, a comparison is not possible, but the value is lower by a factor of five than that of the cubic phase.

**SCHEME 2:**
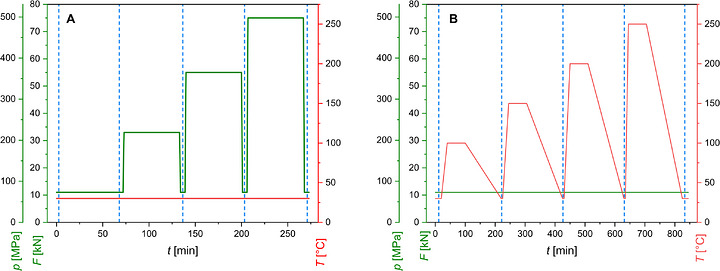
Procedure for pressure treatment up to 664 MPa (A) and temperature treatment up to 250°C (B) with measurement positions dashed in blue. [Correction added on June 2, 2026, after first online publication: Scheme 2 has been updated in this version.]

**FIGURE 2:**
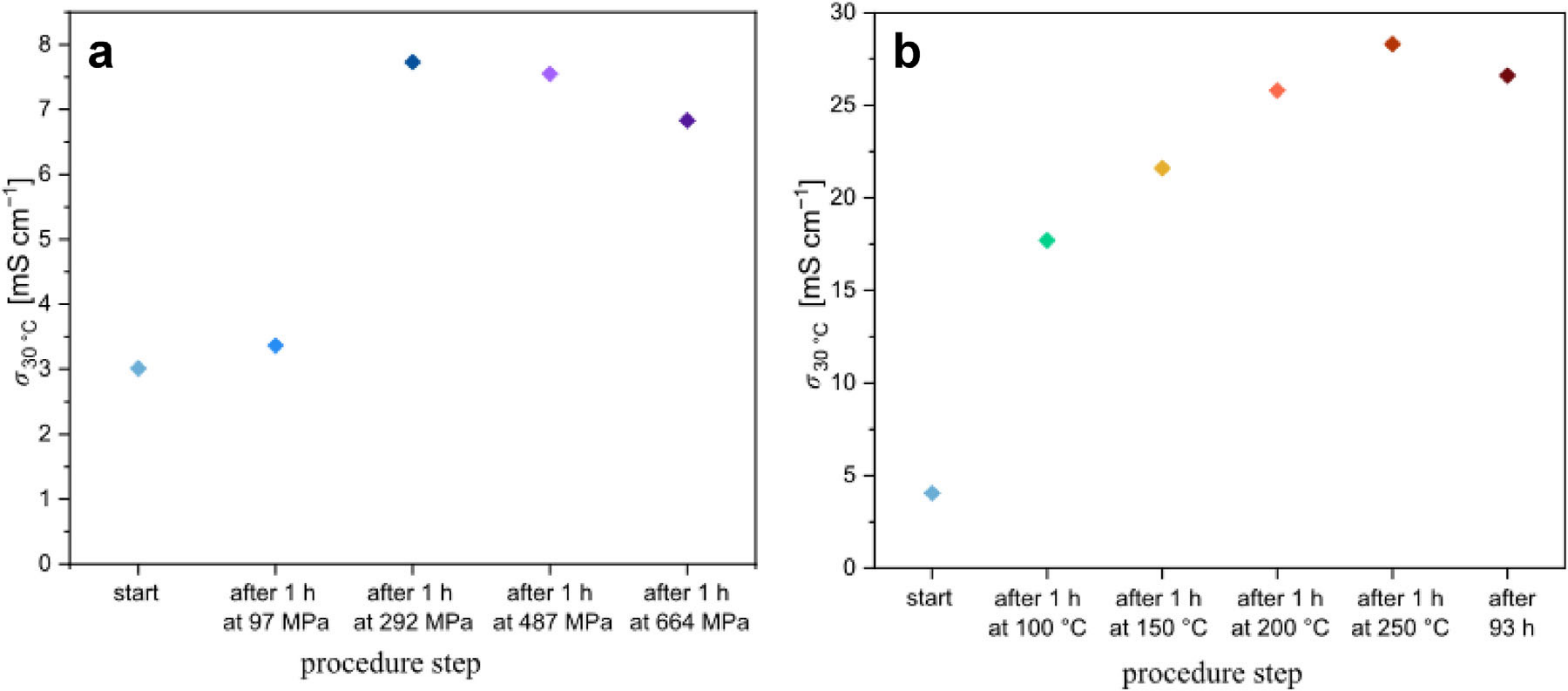
Conductivity of Na_2.9_Sb_0.9_W_0.1_S_4_ after (a) *p*‐ and (b) *T*‐treatment. Impedances were measured at 97 MPa and 30°C.

We used *t*‐Na_2.9_Sb_0.9_W_0.1_S_4_ to investigate systematic ways of pushing ionic conductivities. For this, pressure (*p*‐ramp) and temperature treatments (*T*‐ramp) combined with EIS measurements were investigated (see Experimental Section, Scheme 2). For the *p*‐ramp, an initial EIS measurement was carried out at RT and 97 MPa. Afterward, the pressure was increased and kept constant for 1 h before measuring EIS again at 97 MPa for comparison. Figure 2a shows the resulting conductivities. With this treatment, the ionic conductivity was slightly raised from an initial value of 3.01 to 3.36 mS cm^−1^. The conductivity doubled to 7.73 mS cm^−1^ when the samples were subjected to a pressure of 292 MPa for 1 h. After applying pressures of 487 and 664 MPa, the ionic conductivity decreased slightly to 7.55 and 6.83 mS cm^−1^, respectively, before reaching a plateau. This result was also observed previously in lithium solid electrolytes [8, 9]. The influence of temperature on the ionic conductivity was investigated in a similar way. After an initial EIS measurement at 97 MPa and RT, the temperature was increased stepwise. After each *T*‐step, the temperature was held for 1 h before the sample was allowed to cool down to RTs. The results of the *T*‐ramp study are illustrated in Figure 2b. All EIS measurements were conducted at 97 MPa and RT for comparability. The initial value of 4.8 mS cm^−1^ was nearly quadrupled by treating the sample at 100°C for 1 h. With increasing temperature, *σ* increases linearly, resulting in a conductivity of 28.3 mS cm^−1^ after 1 h at 250°C, which represents the temperature limit of the device. After application of both the *p*‐ramp and *T*‐ramp, the pellets were ground again and investigated by PXRD to study the influence of the treatments on the crystal structure and crystallinity.

Figure S4a shows the PXRD diagrams in comparison with the untreated powder of *t*‐Na_2.9_Sb_0.9_W_0.1_S_4_; prominent reflexes between 28° and 38° are depicted in Figure S4b. PXRD measurements did not indicate a phase transition to *c*‐Na_2.9_Sb_0.9_W_0.1_S_4_ through these treatments, but the *T*‐ramp yielded a slightly higher crystallinity.

Since both treatments led to an increase of the ionic conductivity, although no phase transition to the cubic phase took place, both *p*‐ and *T*‐treatments were combined next. The combination resulted in seven different procedures (denominated as **P1** – **P7**) in which only one parameter was changed at a time (Table 2).

**TABLE 2:** Procedures with varied pressure and temperature parameters, with **P1** being the standard procedure, and **P4** the most efficient one. Changed parameters are highlighted by bold face.

Procedure	*p*‐treatment	*T*‐treatment
**P1**	1 h at 55 kN (487 MPa, 30°C)	1 h at 250°C (11 kN, 97 MPa)
**P2**	**2 h** at 55 kN (487 MPa, 30°C)	1 h at 250°C (11 kN, 97 MPa)
**P3**	1 h at **11 kN** (97 MPa, 30°C)	1 h at 250°C (11 kN, 97 MPa)
**P4**	1 h at **75 kN** (664 MPa, 30°C)	1 h at 250°C (11 kN, 97 MPa)
**P5**	1 h at 55 kN (487 MPa, 30°C)	**2 h** at 250°C (11 kN, 97 MPa)
**P6**	1 h at 55 kN (487 MPa, 30°C)	1 h at **150°C** (11 kN, 97 MPa)
**P7**	1 h at 55 kN (487 MPa, 30°C)	1 h at 250°C (**55 kN, 487 MPa**)

**SCHEME 3:**
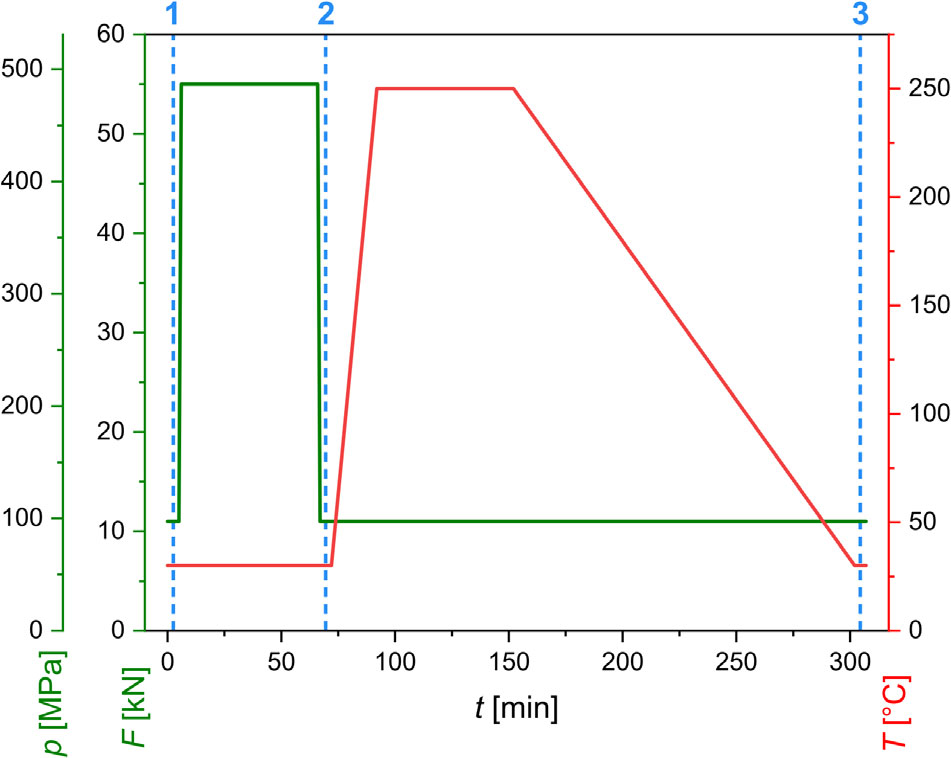
Measuring procedure P1 with initial force/pressure and temperature parameters plotted against time. The three measurements are dashed in blue. [Correction added on June 2, 2026, after first online publication: Scheme 3 has been updated in this version.]

**FIGURE 3:**
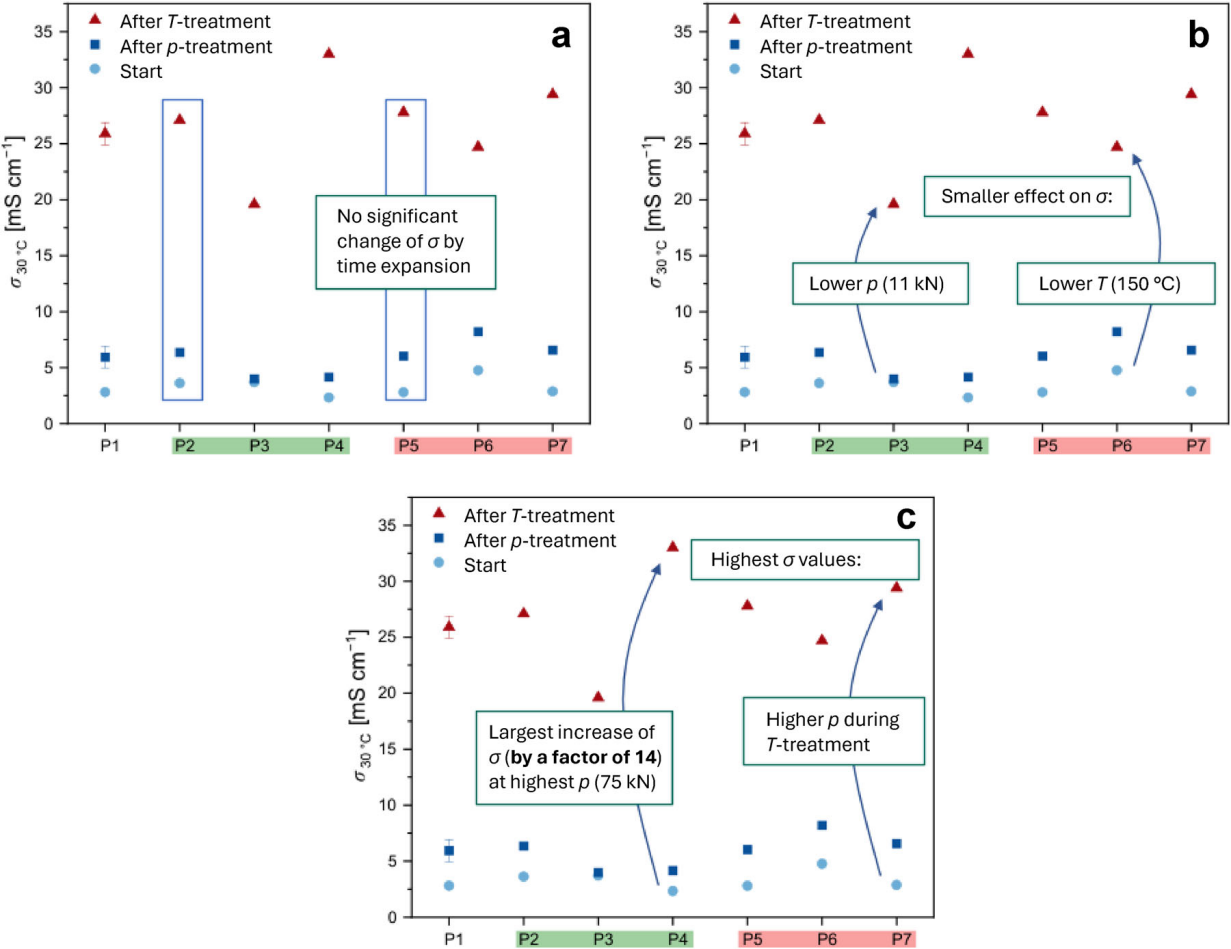
Room temperature conductivities at 97 MPa of the sample prior to any treatment (“Start”) and after the p‐ and T‐treatments of Na_2.9_Sb_0.9_W_0.1_S_4_, pelletized by procedures **P1** to **P7** with error bars shown for three runs of **P1**. The panels highlight different features of the procedures: (a) Measurements after expanding the time of p‐ (**P2**) and T‐treatment (**P5**) to 2 h instead of 1 h. (b) Measurements after treatments at a lower pressure of 97 MPa for the p‐treatment (**P3**) and a lower temperature of 150°C for the T‐treatment (**P6**). (c) Treatments under enhancing the pressure during one of the treatment steps (**P4**, **P7**).

In all cases, the *p*‐treatment was conducted prior to the *T*‐treatment for a given batch of *t*‐Na_2.9_Sb_0.9_W_0.1_S_4_ (5 g). For every procedure, an initial EIS measurement was conducted on the cold‐pressed powder at 97 MPa, and after the *p*‐ and *T*‐treatment at 97 MPa, respectively (see Experimental Section, Scheme 3). The electrochemical performance for all procedures was improved after *p*‐ and *T*‐treatment and resulted in lower real part impedance values, and therefore higher conductivities (Figure S5). Apparently, the resistive contributions from grain boundaries were effectively reduced this way. Procedure **P1** was conducted three times to evaluate the accuracy of the treatments. The resulting ionic conductivity was determined to be 25.9 ± 0.99 mS cm^−1^ after *p*‐ and *T*‐treatment (Figure S6 and Table S1). The calculated conductivities at the starting point, as well as after *p*‐ and *T*‐treatment for all procedures **P1** – **P7**, are shown in Figure 3 and outlined in the following.

Expansion of the time of the *p*‐ (**P2**) and *T*‐treatments (**P5**) to 2 h instead of 1 h did not cause any significant change (Figure 3a). A small raise in conductivity was observed through treatments at lower pressure of 97 MPa (**P3**) and lower temperature of 150°C (**P6**); however, with values of 19.6 mS cm^−1^ (**P3**) and 24.7 mS cm^−1^ (**P6**), the performance was still lower than observed after procedures **P1**, **P2**, and **P5** (Figure 3b). The most promising treatments were those in which the pressure was increased in one of the steps. A conductivity of *σ  =  *29.4 mS cm^−1^ was obtained in procedure **P7** upon raising the pressure during *T*‐treatment to 487 MPa. The largest jump in conductivity, to 33 mS cm^−1^, was detected in procedure **P4**, upon applying 664 MPa (limit of the setup) during the *p*‐treatment (Figure 3c).

Eventually, all procedures served to improve the conductivity of Na_2.9_Sb_0.9_W_0.1_S_4_. Notably, PXRD measured after the pelleting procedures (Figure S7a,c) showed a partial transition from the tetragonal phase to the cubic phase, indicated by (partial) vanishing of the splitting of reflections typical for the tetragonal phase (see Figure S7b,d for prominent peaks). The occurrence of *c*‐Na_2.9_Sb_0.9_W_0.1_S_4_ goes hand in hand with the observed increase in conductivity.

Diffusion coefficients *D*(Na^+^) were calculated from values for *σ*_30°C_, for all procedures, according to the *Nernst‐Einstein* equation (see Table S2). The highest value of *D*(Na^+^) = 3.26 10^−11^ m^2^ s^−1^ was achieved after **P4**, in agreement with the conductivity values. Raman spectroscopy reveals no change in the vibrational modes before and after **P4** (Figure S8), indicating the sulfido metalate tetrahedra are not affected at the atomic scale by the *p‐* and *T*‐treatments.

**SCHEME 4:**
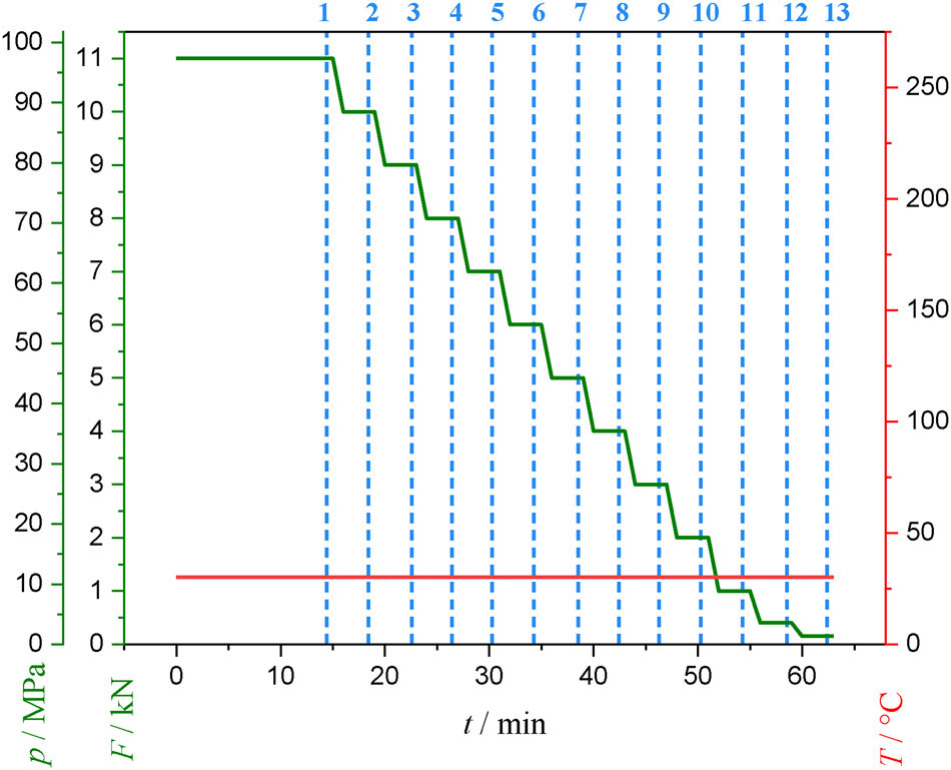
Procedure for pressure ramp to low pressures with measurement positions dashed in blue.

**FIGURE 4:**
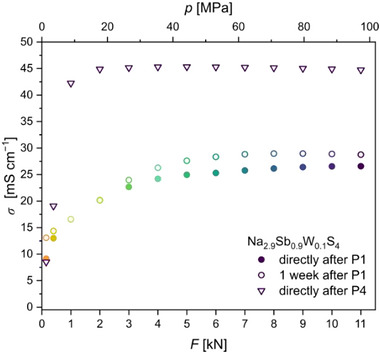
Conductivities measured after application of pressure ramps from 97 to 1.3 MPa of Na_2.9_Sb_0.9_W_0.1_S_4_. The measurements were conducted directly after termination of procedures **P1** (filled circles) and **P4** (open triangles), as well as one week after terminating **P1** (open circles).

Since most of the reported conductivities are only valid for the high pressures (above 320 MPa) applied during EIS measurements, we further examined the low‐pressure performance of Na_2.9_Sb_0.9_W_0.1_S_4_ after the procedures were terminated. This is particularly important in terms of the materials’ application potentials, as high stack pressures cannot be realized in industrial‐scale batteries. Here, the requirement is a pressure of less than 1 MPa [17]. Due to setup limitations, a minimum pressure of 1.3 MPa was applied. Downward pressure ramps (down to 1.3 MPa, see Experimental Section, Scheme 4) were carried out after the initial procedure, **P1**, and after the most efficient procedure, **P4**. Figure 4 illustrates the results upon application of these pressure ramps. Corresponding EIS measurements are shown in Figure S9.

For **P1**, the pressure ramp was measured directly after the described treatment, and also after keeping the sample treated according to **P1** at 97 MPa for one week. The results of the first *p*‐ramp measurements indicate that the initial conductivity of 26.6 mS cm^−1^ at 97 MPa steadily decreases as the pressure drops. The conductivity decreases in small steps down to a pressure of 44 MPa and declines more significantly below this pressure. Nevertheless, the finally reached conductivity still amounts to 9.1 mS cm^−1^ at 1.3 MPa. Notably, the *p*‐ramp measured one week after carrying out procedure **P1** shows an even higher final conductivity at low pressures. Starting at an initial value of 28.8 mS cm^−1^ at 97 MPa, the conductivity remains at an excellent value of 13.1 mS cm^−1^ at 1.3 MPa. We attribute this observation to further compaction upon the impact of a constant pressure of 97 MPa during one week. The same procedure applied to the sample following procedure **P4**, a record superionic conductivity of 44.7 mS cm^–^^1^ was measured, slightly higher than the reported value of 41 ± 8 mS cm^−1^. During the *p*‐ramp down to low pressures, this conductivity held up steady down to a pressure of 18 MPa. Afterward a drop in conductivity occurred that led to a low‐pressure conductivity of 8.4 mS cm^−1^ at 1.3 MPa, similar to the one observed upon lowering the pressure directly after procedure **P1**.

FIB‐SEM allowed more insight into the effect of applying procedure **P4** on the microstructure on the pellets. A pellet obtained from powder cold‐compressed at 487 MPa for 1 h without any further treatment was therefore compared with a pellet after conducting **P4**. Overview images of the polished cross sections are shown in Figure S10A,B. Generally, the images indicate a good connection of the particles. However, while the ‘untreated’ pellet is rather uneven and possesses large voids, especially in the surface regions, the cross‐section of the pellet after application of **P4** is very smooth and much more even. Detailed views of the cross‐section of the “untreated” versus the “treated” pellet (Figure S10C,D, respectively) highlight that the application of **P4** affords a very homogenous particle connection, which results in a dense pellet exhibiting smaller and fewer void spaces.

## 3 Conclusion

The development of ion conductivities of tetragonal and cubic phases of Na_2.9_Sb_0.9_W_0.1_S_4,_ *t*‐Na_2.9_Sb_0.9_W_0.1_S_4_, and *c*‐Na_2.9_Sb_0.9_W_0.1_S_4_, was investigated by systematic application of pressure and temperature. First, *p*‐ and *T*‐treatments were studied separately. *T*‐treatments had a greater effect in terms of improving the ionic conductivity, although the phase transition of *t*‐Na_2.9_Sb_0.9_W_0.1_S_4_ to *c*‐Na_2.9_Sb_0.9_W_0.1_S_4_ was not detectable by PXRD. Next, we developed and analyzed seven procedures for the combination of pressure and temperature treatments (**P1** – **P4**) in which only one parameter (*p*, *T*, *t*) was changed at a time. The highest ionic conductivity value in this series, 33 mS cm^−1^ at 97 MPa, was achieved by pressurizing the sample for 1 h at 664 MPa and subsequently applying a temperature of 250°C (at 97 MPa) for 1 h (**P4**). All procedures led to (at least partial) transformation into the cubic phase. The low‐pressure performance after a downward *p*‐ramp applied to a sample after consecutive 1 h *p*‐ and *T*‐treatments at (1) 487 MPa and (2) 250°C at 97 MPa (**P1**) showed very high remaining ionic conductivities of 9.1 mS cm^−1^ upon relaxation to 1.3 MPa. After exposure of a **P1** sample to 97 MPa for one week, measurement of the conductivity revealed an even higher value of 13.1 mS cm^−1^. The same study carried out for a sample after procedure **P4** afforded a record conductivity value for *c*‐Na_2.9_Sb_0.9_W_0.1_S_4_ of 44.7 mS cm^−1^, which persisted during relaxation of the pressure down to 18 MPa, and reached at an excellent value of 8.4 mS cm^−1^ at 1.3 MPa. FIB‐SEM imaging revealed significant compaction of the SE pellet following **P4**, underscoring the positive effect of the *p*‐ and *T*‐treatments. The results reported herein demonstrate realistic avenues for the application of *c*‐Na_2.9_Sb_0.9_W_0.1_S_4_ in solid‐state batteries and showcase how to ensure a good solid electrolyte performance at the low pressures required for industry‐scale batteries.

## 4 Experimental Section/Methods

### 4.1 General Information and Materials

All synthesis and analytical preparations were carried out in a glovebox under a dry argon atmosphere. The starting material Na_2_S was prepared by using the known procedure in liquid NH_3_ from the elements [19]. The starting material WS_2_ was prepared by solid‐state reaction from the elements and is described elsewhere [19].

### 4.2 Synthesis of Sodium Solid Electrolytes

All sulfide‐based sodium solid electrolytes were synthesized by a solid‐state reaction. For Na_3_SbS_4_, the starting materials Na_2_S, Sb (Sigma–Aldrich, 99 %), and S (Thermo Fisher, 99 %) were used. For Na_2.9_Sb_0.9_W_0.1_S_4_, the as prepared WS_2_ was added. The stoichiometric amounts of starting materials were ground in an agate mortar and filled into fused silica tubes, respectively. The tubes were transferred to the SCHLENK‐line, evacuated, and sealed by means of a propane/O_2_ burner. Next, the tubes were loaded into to a chamber furnace, heated to 550°C with a heating rate of 30°C/h, hold for 20 h, and cooled to room temperature by quenching in an iced water bath (Scheme 1). The products Na_3_SbS_4_ and Na_2.9_Sb_0.9_W_0.1_S_4_ were obtained as green powders.

### 4.3 X‐Ray Powder Diffraction Measurements

Powder diffraction patterns were collected on a STADI MP (STOE, Darmstadt) powder diffractometer equipped with a MYTHEN 1K detector using mirror monochromated Mo‐*K_α_* radiation (*λ*  =  0.72 Å) or Cu‐*K_α_* radiation (*λ*  =  1.54 Å). The samples were ground in an agate mortar, filled into capillaries (0.4 mm), and measured in the Debye‐Scherrer geometry in an angle range from 5° to 35°.

### 4.4 Electrochemical Impedance Spectroscopy

For measuring the SSE powder a CompreDrive measuring stand (RHD instruments) was used. For comparability, 130 mg of the grounded solid electrolyte powders were weighed, filled into the measuring cell (RHD instruments, double piston cell with Alox (Al_2_O_3_) inlay, 12 mm), distributed evenly, and put into the measuring stand. Temperature‐dependent EIS was measured between 30°C and 85°C at 389 MPa. Besides that, Different pressure and temperature profiles were used in this study, which are described later. However, for all the measurements after convergence of the temperature and pressure parameters, EIS was measured by applying an alternating potential of 10 mV in a frequency range of 10^5^–10 Hz. [Correction added on June 2, 2026, after first online publication: The term “10510 Hz” has been corrected to “10^5^–10 Hz” in this version.] Impedance spectra were analyzed and fitted with the RelaxIS software (rhd instruments) using the high‐frequency modulus. All spectra were fitted by using a resistor *R* and a constant phase element *C*_CPE_ in series as an equivalent circuit. The obtained values for the resistance *R*, together with the pellet thickness *d* and area *A* were used to calculate the conductivities by applying Equation (1):

(1) $$\sigma =\frac{1}{R}\cdot \frac{d}{A}$$

### 4.5 Pressure and Temperature Procedures

The pressure (*p*‐treatment) and temperature ramp (*T*‐treatment) procedures were developed to investigate the effect on the conductivity and possible phase changes of the compound. For the *p*‐treatment, the temperature was kept constant at 30°C, while the force on the pellet was increased in steps of 11 kN (97 MPa), 33 kN (292 MPa), 55 kN (487 MPa), and 75 kN (664 MPa; setup limitation), see Scheme 2a. Each pressure step was held for a duration of 1 h. For the *T*‐treatment, the force on the pellet was kept constant at 11 kN, while the temperature was increased in steps to 100°C, 150°C, and 200°C up to the setup limitation of 250°C (Scheme 2b). Each heating step was conducted for a duration of 1 h. After each step and cooling down naturally, EIS was conducted as described above.

The influence of combining temperature and pressure on the conductivity was investigated by varying the thermo‐mechanical parameters as part of a newly developed procedure. In order to separate the pressure effect from the temperature effect, the measuring procedure was divided into two steps: *p*‐treatment in the first step and *T*‐treatment in the second step. Cooling down after *T*‐treatment occurred naturally. EIS measurements were carried out before and after the procedure, as well as in between the two steps. All three measurements took place at 30°C and 11 kN (97 MPa) for comparability reasons (Scheme 3).

In further steps, force and temperature parameters of the procedures were varied, while the measurements were conducted at constant force and temperature (11 kN and 30°C). Thermo‐mechanical parameters for the standard procedure (**P1**) as well as for the varied procedures (**P2** – **P7**) are given in Table 2.

Conductivities at low pressures were measured by reducing the force stepwise, starting from 11 kN down to the setup limit of 0.15 kN as depicted in Scheme 4. Impedance spectra for each step were measured at 30°C This measuring procedure was performed on Na_2.9_Sb_0.9_W_0.1_S_4_ directly after **P1** to measure the low‐pressure performance after the pellet preparation by **P1**. The procedure was repeated after one week at 30°C and 11 kN to examine the effect of time on the low‐pressure performance.

### 4.6 Raman Spectroscopy

Raman spectra were collected using a Renishaw inVia confocal Raman spectrometer with a 532 nm laser, diffraction gratings (532 nm), and an ultrahigh sensitivity CCD detector. An objective lens with 40x magnification was used to focus the incident beam, and a filter was used to reduce the laser power to 0.5 % of the total power. For sample preparation, untreated Na_2.9_Sb_0.9_W_0.1_S_4_ powder and powder pellet of procedure P4 were put on a microscopy slide and covered with crystallography oil to ensure that the sample was protected from air/moisture. Samples were measured in a wavenumber range from 1000 to 100 cm^−1^ at room temperature using the WiRE software with a spectral detector resolution of 0.5 cm^−1^.

### 4.7 Scanning Electron Microscopy (SEM) and Focused Ion Beam (FIB)

Cross sections of Na_2.9_Sb_0.9_W_0.1_S_4_ pellets were taken by using a Tescan FIB‐SEM system with an Xe plasma FIB. For milling of the cross‐section, a potential of 30 keV and a current of 2.3 µA was used. For polishing of the cross‐section, the current was reduced to 100 nA. Images of the cross‐section were taken with the Axial + MD detector of the device.

## Conflicts of Interest

The authors declare no conflicts of interest.

## Supporting information




**Supporting File**: smll73724‐sup‐0001‐SuppMat.pdf.

## Data Availability

The data that support the findings of this study are available from the corresponding author upon reasonable request.
