# Supplementary material for: Super‐High Sodium‐Ion Conductivity of Na2.9Sb0.9W0.1S4 at Low Pressures by Systematic Pressure and Temperature Treatments
Source: Small. 2026 May 10;22(36):e00075. doi: 10.1002/smll.202600075 (PMC13307257; doi:10.1002/smll.202600075)
Supplement: Supplementary file 1 — Supporting File: smll73724‐sup‐0001‐SuppMat.pdf. [file SMLL-22-e00075-s001.pdf]

Supporting Information

Super-high sodium-ion conductivity of $Na_{2.9}Sb_{0.9}W_{0.1}S_4$ at low pressures through systematic pressure and temperature treatments

*Miriam R. Bauer, Marvin Szabo, Stefanie Dehnen**

Institute of Nanotechnology (INT), Karlsruhe Institute of Technology (KIT), Kaiserstraße 12, 76131 Karlsruhe, Germany

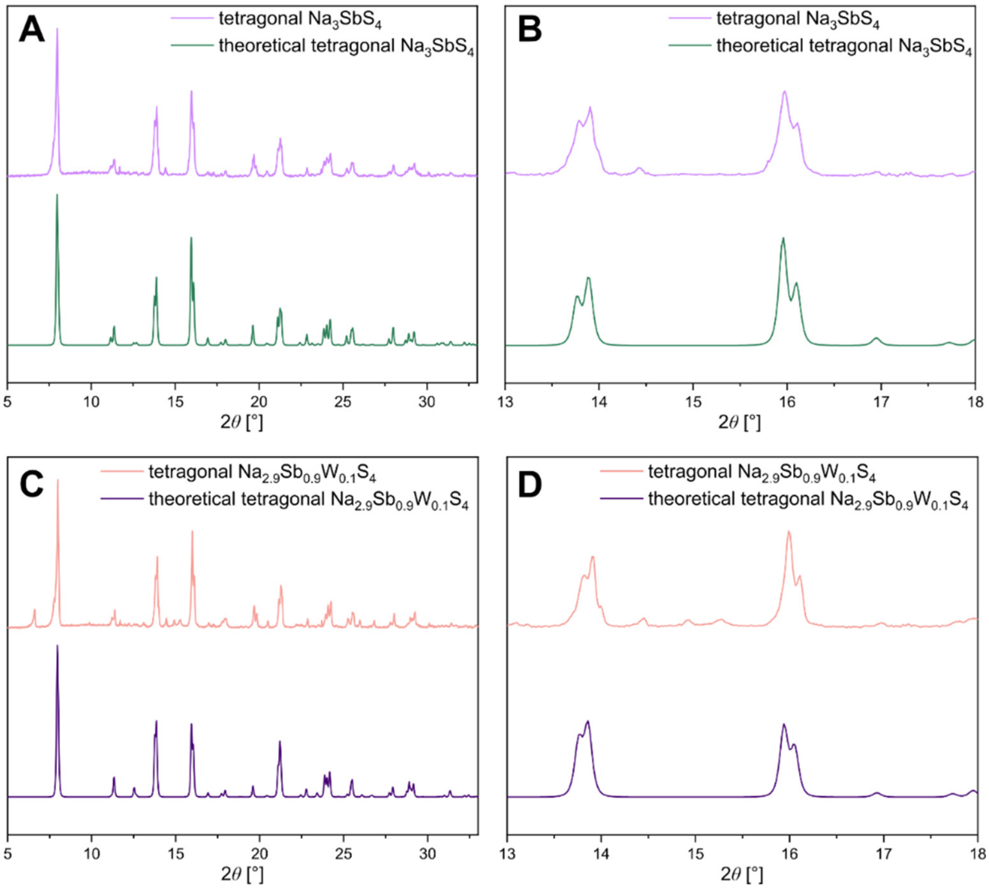

Figure S1: Powder X-Ray diffractograms (PXRD) of Na_3SbS_4 (A, B) and $Na_{2.9}Sb_{0.9}W_{0.1}S_4$ (C, D) in comparison with theoretical diffractograms of tetragonal Na_3SbS_4 and $Na_{2.9}Sb_{0.9}W_{0.1}S_4$.[1,2] (B) and (D) show the prominent reflexes between 13° and 18° (Mo-K_α).

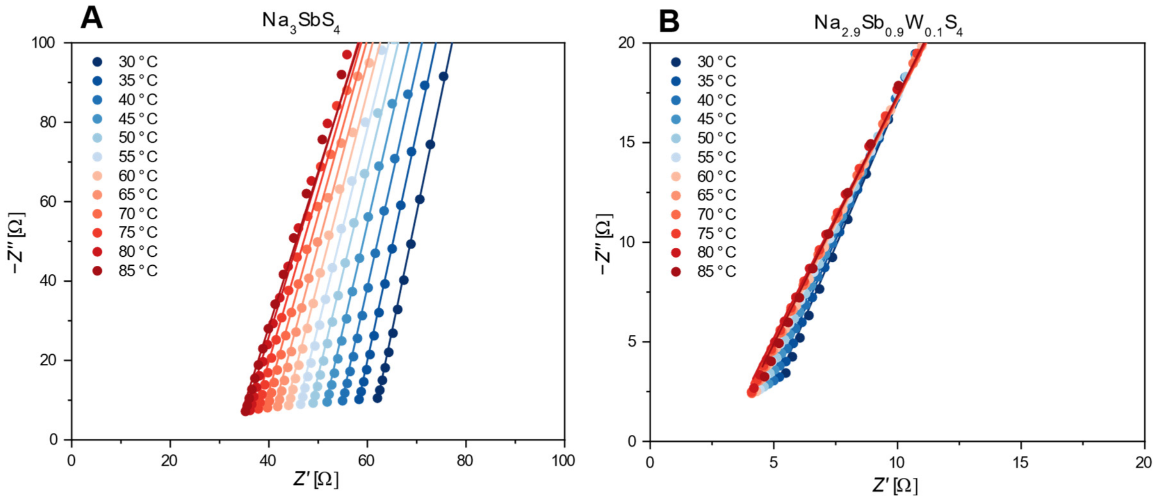

Figure S2 Impedance spectra of Na_3SbS_4 (A) and $Na_{2.9}Sb_{0.9}W_{0.1}S_4$ (B) measured from 30 °C to 85 °C. The impedances were fitted for each temperature using a R-C_{CPE} circuit model.

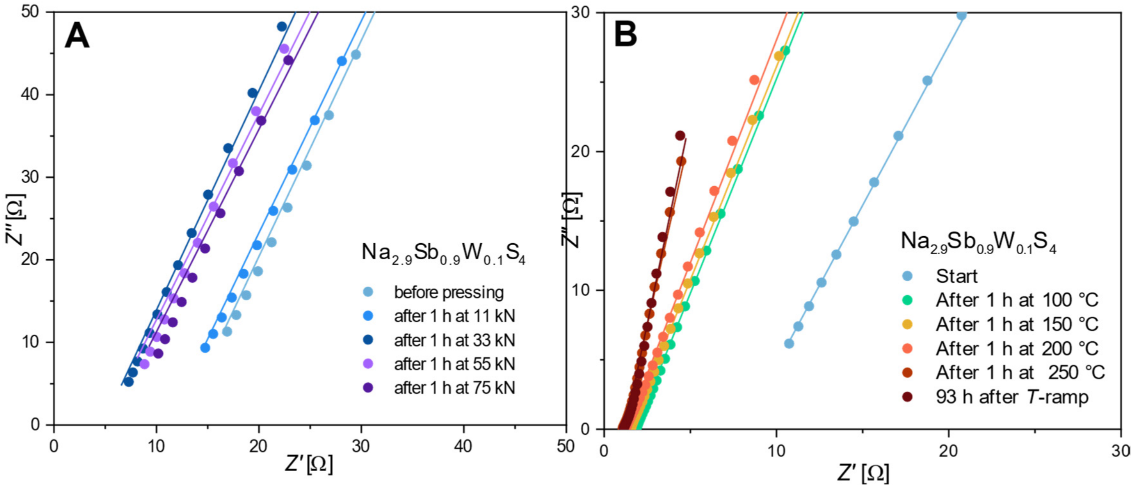

Figure S3: Impedance spectra of *p*- (A) and *T*-treatment (B) measurements fitted with a R-C_{CPE} equivalent circuit model. EIS were measured after every treatment step at 11 kN and 30 °C.

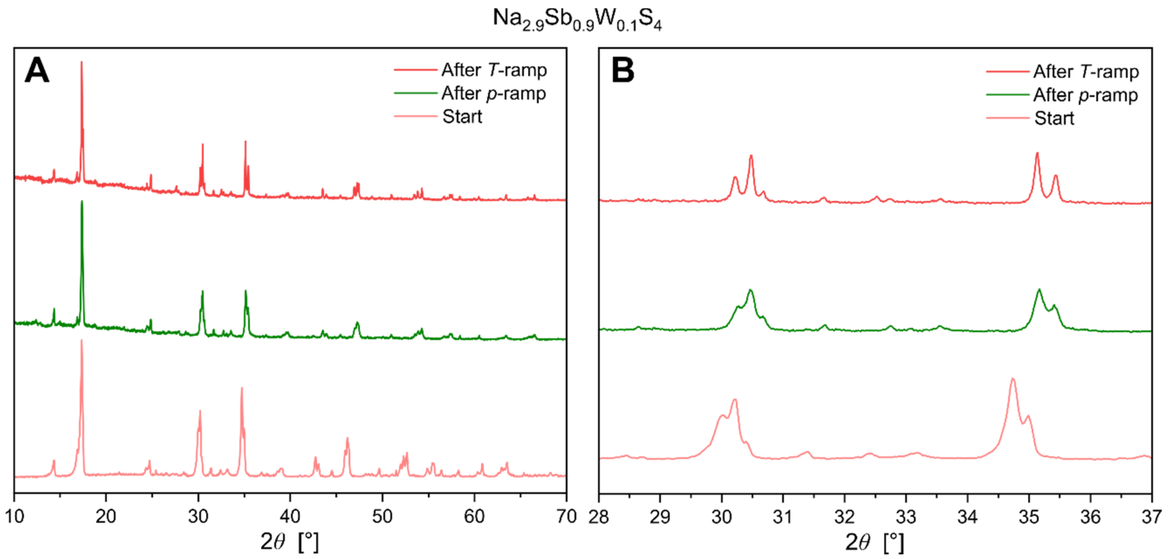

Figure S4 Powder X-Ray diffractograms of a $Na_{2.9}Sb_{0.9}W_{0.1}S_4$ pellet prepared by *p*- and *T*-treatment. PXRD of the starting powder used for pellet pressing was measured with Mo-K_α source and shifted for comparison. (B) only shows the prominent reflexes between 28° and 37° (Cu-K_α).

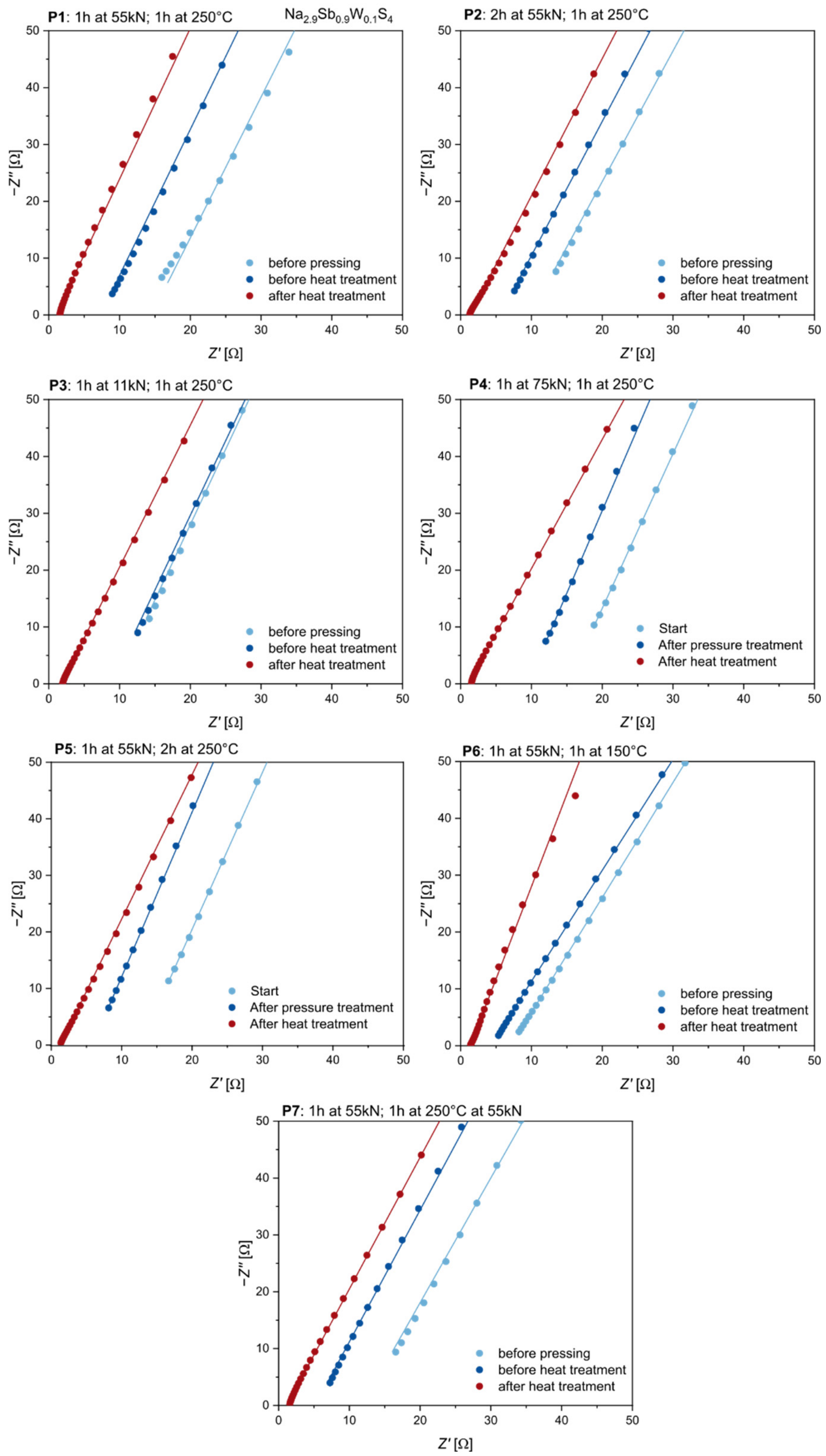

Figure S5: Nyquist plots of $Na_{2.9}Sb_{0.9}W_{0.1}S_4$ pellets prepared by P1–P7, all including variations in pressure treatment. Impedances were measured at 11 kN and 30 °C. The data was fitted using a R-C_{CPE} circuit model.

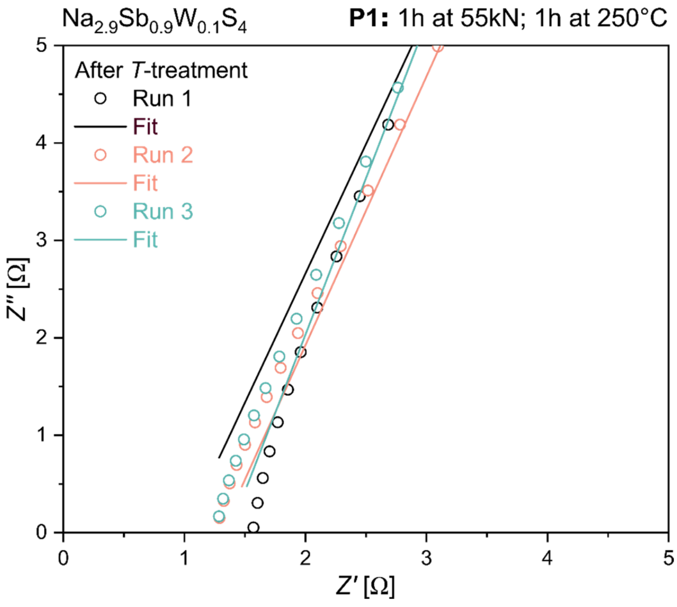

Figure S6: Impedance spectra after *T*-treatment of $Na_{2.9}Sb_{0.9}W_{0.1}S_4$ pelletized by procedure P1: Run 1, 2 and 3. Impedances were measured at 11 kN and 30 °C. The data was fitted using a *R*-C_{CPE} circuit model.

Table S1: Mean and standard deviation calculated from $\sigma_{30\,°C}$ of SE pellets made of tetragonal $Na_{2.9}Sb_{0.9}W_{0.1}S_4$. The value in bold results from an inaccurate fit and is disregarded for further calculations.

P1 (1 h at 55 kN; 1 h at 250 °C)	$Na_{2.9}Sb_{0.9}W_{0.1}S_4$ $\sigma_{30\,°C}$ [mS cm^{-1}]			Mean [mS cm^{-1}]	Standard deviation [mS cm^{-1}]
	Run 1	Run 2	Run 3		
Start	2.73	2.80	2.90	2.81	0.09
After *p*-treatment	4.95	6.00	6.85	5.95	0.97
After *T*-treatment	~~36.2~~	26.6	25.2	25.9	0.99

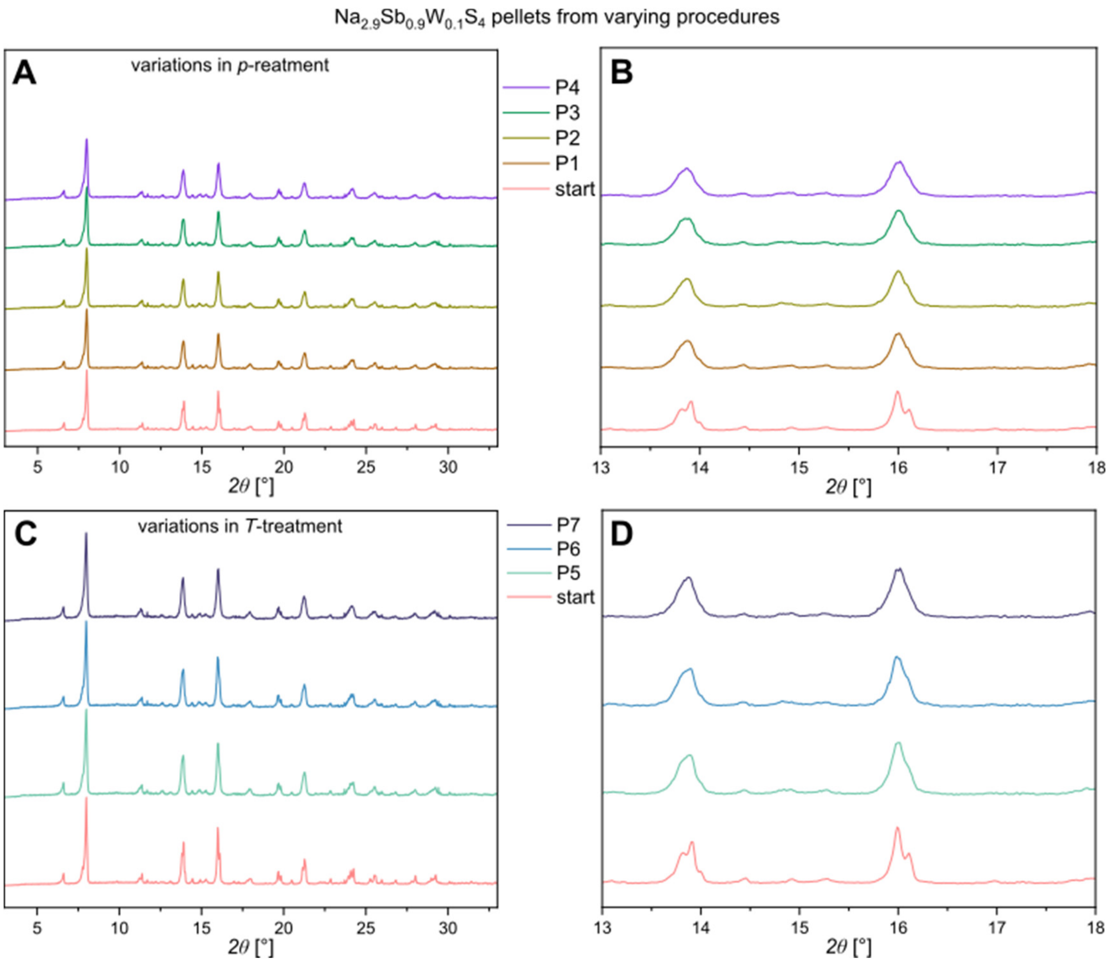

Figure S7: Powder X-Ray diffractograms of pellets prepared by procedures P1–P7, compared with the $Na_{2.9}Sb_{0.9}W_{0.1}S_4$ powder used for pellet pressing. (A) and (B) show the diffractograms of pellets prepared by procedure P1–P4 with variations in the *p*-treatment. (C) and (D) show the diffractograms of pellets prepared by procedure P5–P7 with variations in the *T*-treatment. (B) and (D) only show the prominent reflexes between 13° and 18° (Mo-K_{α}).

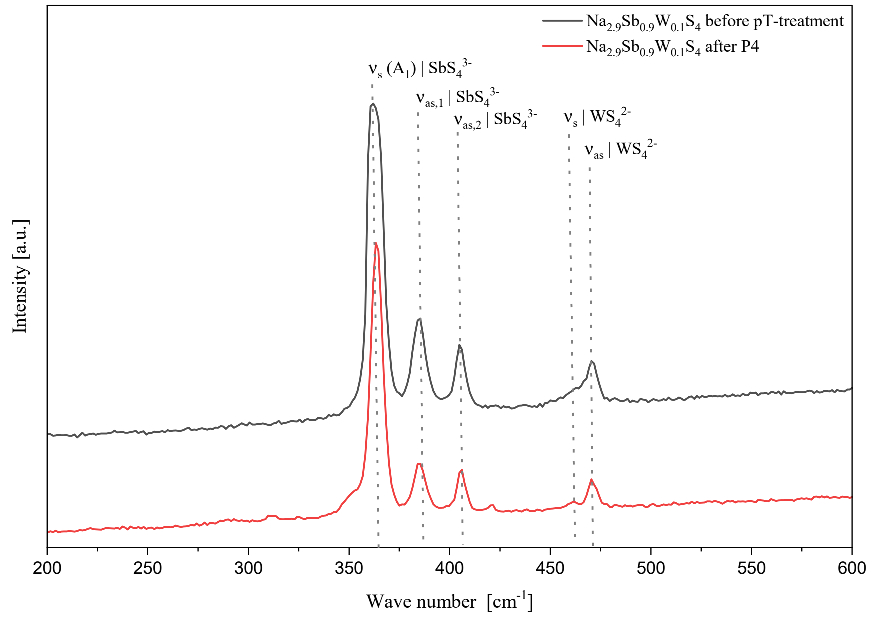

Figure S8: Raman spectra of $Na_{2.9}Sb_{0.9}W_{0.1}S_4$ before (black) and after (red) conducting procedure P4. Raman signals can be attributed to the different modes of $[SbS_4]^{3-}$ [2,3] and $[WS_4]^{2-}$ [4] tetrahedra.

Table S2: Ionic conductivities at 30 °C, $\sigma_{30°C}$, measured at the start, after *p*-treatment, and after *T*-treatment of procedures **P1** – **P7**, and corresponding diffusion coefficients $D(Na^+)$ calculated with the Nernst-Einstein equation, $D(Na^+) = \frac{\sigma_{30°C}\, k_B T}{n q^2}$, from experimental $\sigma_{30°C}$ values; k_B = Boltzmann constant, T = 303.15 K, $n = 1.65 \cdot 10^{28}$ (number of Na^+ ions per volume), $q = 1.602 \cdot 10^{-19}$ C (charge of Na^+ ions); the correlation factor is assumed to be $f = 1$.

Procedure	Ionic Conductivity $\sigma_{30\,°C}$ [mS cm^{-1}]			Diffusion Coefficient $D(Na^+)$ [m^2 s^{-1}]		
	start	After *p*-treatment	After *T*-treatment	start	After *p*-treatment	After *T*-treatment
P1	2.81	5.95	25.9	$2.78 \cdot 10^{-12}$	$5.88 \cdot 10^{-12}$	$2.56 \cdot 10^{-11}$
P2	3.62	6.36	27.1	$3.58 \cdot 10^{-12}$	$6.28 \cdot 10^{-12}$	$2.68 \cdot 10^{-11}$
P3	3.70	4.0	19.6	$3.66 \cdot 10^{-12}$	$3.95 \cdot 10^{-12}$	$1.94 \cdot 10^{-11}$
P4	2.34	3.73	33.0	$2.31 \cdot 10^{-12}$	$3.68 \cdot 10^{-12}$	$3.26 \cdot 10^{-11}$
P5	2.80	6.04	27.8	$2.77 \cdot 10^{-12}$	$5.97 \cdot 10^{-12}$	$2.75 \cdot 10^{-11}$
P6	4.77	8.21	24.7	$4.71 \cdot 10^{-12}$	$8.11 \cdot 10^{-12}$	$2.44 \cdot 10^{-11}$
P7	2.88	6.57	29.4	$2.85 \cdot 10^{-12}$	$6.49 \cdot 10^{-12}$	$2.9 \cdot 10^{-11}$

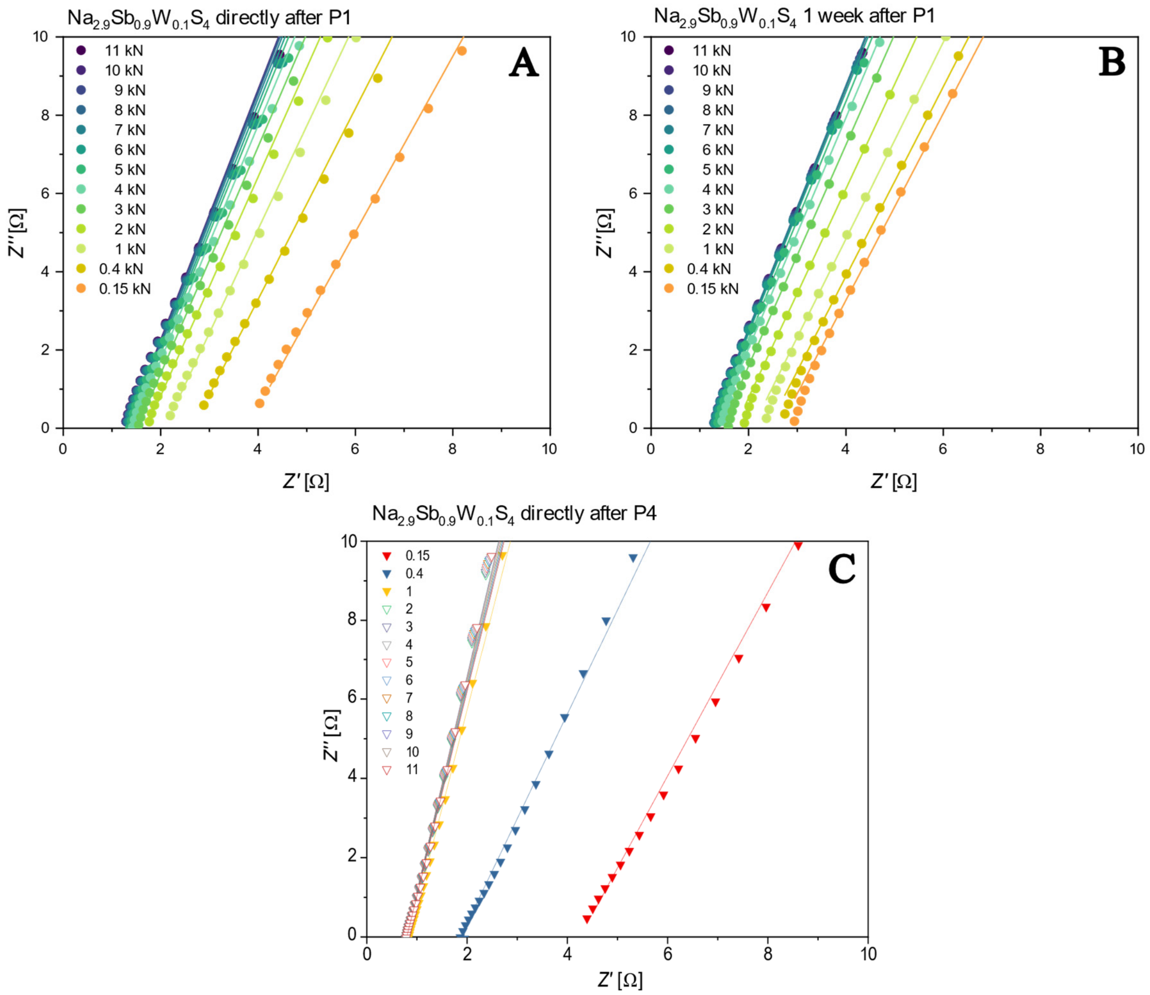

Figure S9: EIS measurements from pressure ramps from 97 MPa to 1.3 MPa of $Na_{2.9}Sb_{0.9}W_{0.1}S_4$. The measurements were conducted directly after procedure P1 (A) and P4 (C) and additionally one week after P1 (B).

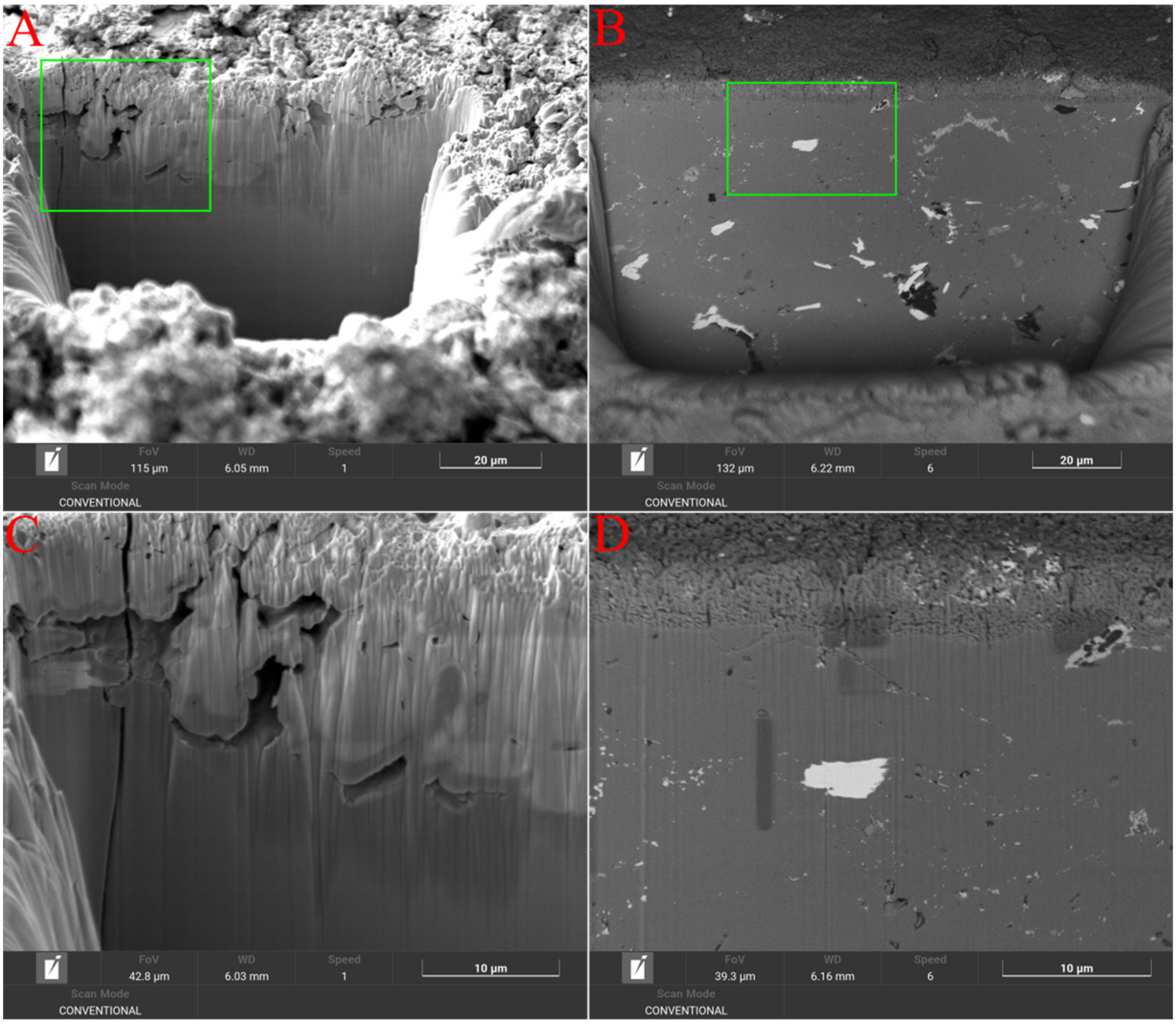

Figure S10: Polished cross sections of $Na_{2.9}Sb_{0.9}W_{0.1}S_4$ pellets prepared by compression at 487 MPa before applying p- and T-treatment (A, C) and after conducting procedure P4 (B,D). A and B represent overview images, C and D show zooms into the areas marked with a green rectangle in A and B, respectively. The change towards lower porosity after procedure P4 is obvious.

References

[S1] O. Maus, M. T. Agne, T. Fuchs, P. S. Till, B. r. Wankmiller, J. M. Gerdes, R. Sharma, M. Heere, N. Jalarvo, and O. Yaffe, *On the discrepancy between local and average structure in the Fast Na$^+$ ionic conductor $Na_{2.9}Sb_{0.9}W_{0.1}S_4$*, *J. Am. Chem. Soc.* **2023**, 145, 7147–7158.

[S2] A. Banerjee, K. H. Park, J. W. Heo, Y. J. Nam, C. K. Moon, S. M. Oh, S. T. Hong, and Y. S. Jung, *Na_3SbS_4: a solution processable sodium superionic conductor for all-solid-state sodium-ion batteries*, *Angew. Chem. Int. Ed.* **2016**, *55*, 9634–9638.

[S3] W. Mikenda, A. Preisinger, *A vibrational spectra of Na_3SbS_4 Na_3SbS_4*H_2O (Schlippe's Salt) and Na_3SbS_4*$9D_2O$. Spectrochim. Acta Part A Mol. Spectrosc.* **1980**, *36*, 365–370.

[S4] A. Müller, W. Jaegermann, W. Hellmann, *Schwingungsspektren und Strukturchemie von Heterometall-Komplexen und Clustern mit Thiometallat-Liganden, J. Mol. Struct.* **1983**, *100*, 559–570.
